# Prevalence and Perceptions of Musculoskeletal Disorders Among Hospital Nurses in Pakistan: A Cross-sectional Survey

**DOI:** 10.7759/cureus.1001

**Published:** 2017-01-26

**Authors:** Farooq A Rathore, Rayan Attique, Yumna Asmaa

**Affiliations:** 1 Department of Rehabilitation Medicine, PNS Shifa Hospital, DHA II, Karachi 75500, Pakistan; 2 Department of Rehabilitation Medicine, Bahria University Medical and Dental College, Bahria University, DHA -II, Karachi; 3 Department of Rehabilitation Medicine, Faculty of Rehabilitation Medicine, University of Alberta, Edmonton, Canada; 4 Department of Rehabilitation Medicine, CMH Lahore; 5 Department of Medicine, Combined Military Hospital, Lahore, Pakistan

**Keywords:** nursing, nurses, work related musculoskeletal disorders, ergonomics, ergonomics in nursing, back pain, neck pain, pakistan, disease prevention, cumulative trauma disorders

## Abstract

**Introduction:**

Nursing is a professionally demanding job, and nurses are prone to develop musculoskeletal disorders. However, no data is available regarding its prevalence among Pakistani nurses. This study was conducted to document the pattern of work-related musculoskeletal disorders (WRMDs) in Pakistani nurses and their perceptions about contributing factors and management of WRMDs.

**Methods:**

A questionnaire-based, cross-sectional survey was conducted in six hospitals in Lahore and Rawalpindi, which were selected using a convenient sampling technique. A four-part questionnaire comprised of demographic data, experience of musculoskeletal disorders, and perception of management and contributing factors of WRMDs was distributed among 150 nurses. One hundred and seventeen nurses returned completed forms. Data was analyzed using SPSS Statistics v20 (IBM, Armonk, New York, USA).

Ethics review committee approval was obtained by CMH Lahore Medical College and the Institute of Dentistry, and informed consent was obtained.

**Results:**

The prevalence of musculoskeletal disorders over a 12-month period was 31.6%, with the most common site being the low back (32%) followed by the shoulder (20%), upper back, and knees (10%). Among those affected, 60.6% sought professional help. Married nurses were more prone to WRMDs (p=0.0001). Regarding management, most (94%) agreed that rest is required to get better, neglecting problems of this kind can cause permanent health problems (89.7%), and physical activity should be avoided (38.7%). Working in the same positions for long periods (93.1%), attending an excessive number of patients in one day (81.2%), and working in awkward and cramped positions (78.6%) were the most commonly perceived risk factors for WRMDs.

**Conclusion:**

About one-third of Pakistani nurses in this cohort reported work-related musculoskeletal disorders with the low back most commonly affected. There is a need to increase awareness regarding ergonomics and posture maintenance to reduce WRMDs and improve patient care. This can be achieved by workshops and seminars on ergonomics and WRMDS.

## Introduction

The World Health Organization (WHO) defines WRMDs as “health problems of the locomotor apparatus, i.e. muscles, tendons, the skeleton, cartilage, ligaments and nerves.” It also notes that WRMDs can be “caused or intensified by work, though often activities such as housework or sports may also be involved” [1]. WRMDs rank high on the list of health disorders commonly affecting humans. From 2014-2015 in Great Britain, about 9.5 million working days were lost because of WRMDs, accounting for 40% of working days lost due to ill health [2].

Musculoskeletal pain has been reported in computer users [3], office workers [4], ophthalmologists [5], professional musicians [6], and marines [7]. Medical professionals are among the most frequently affected by WRMDs. Surgeons are most commonly affected, followed by nurses and physiotherapists [8]. Nurses are an integral part of the healthcare team, as they bridge the gap between doctors and patients, and facilitate healthcare delivery in hospitals. Because of the nature of their work, nurses are prone to WRMDs. Studies in Nigeria [9-10], Turkey [11], Australia [12], Estonia [13], and Japan [14] have shown the average prevalence of WRMDs among nurses to be about 84%, with the lower back most commonly reported as the site involved. A cohort-based study among Taiwanese nurses also noted a trend of increasing incidence of WRMDs from 2005 (28.45%) to 2010 (33.65%) [15], emphasizing the importance of the subject.

There is no published data on WRMDs in Pakistani nurses. According to the 2013 estimates, the nurse-to-patient ratio in Pakistan was 1:50, whereas the ratio prescribed by the Pakistan Nursing Council is targeted at 1:10 in general areas and 1:2 in specialized areas [16]. This is much higher than the ratio in developed countries. Therefore, a higher rate of WRMDs in Pakistani nurses can be anticipated. Unfortunately, this issue has received little attention in developing countries like Pakistan. Possible reasons can be a lack of interest and awareness regarding the topic,  ignorance among paramedical staff including nurses, lack of resources and funds for researchers, and a communication gap between researchers and the target audience.

We conducted this study with two aims: 1) to document the pattern of WRMDs in Pakistani nurses; and 2) to explore perceptions of nurses toward contributory factors and prevention of WRMDs.

## Materials and methods

Approval was obtained from the ethics review committee of CMH Lahore Medical College and the Institute of Dentistry. We used the questionnaire by Tinubu et al. [10], after obtaining formal permission from the corresponding author. We modified it considering the local work environment of Pakistan. Postgraduate education of all healthcare professionals in Pakistan including nurses is in English. Therefore, the English version was used without any translation into the local language. The first section was an informed consent form. It explained the rationale and possible benefits of the study and assured anonymity of the data and personal identification. The second section had demographics and included questions on the pattern, location, and duration of WRMDs. The third section explored views on causes and prevention of pain. The fourth section documented perceptions of work-related risk factors that may contribute to the development of WRMDs. We conducted a pilot survey, and based on the feedback, removed two items to adapt the questionnaire content to the Pakistani culture and work environment. To facilitate easier responses, it was further modified from a zero-to-10 scale to a five-point, Likert-type scale consisting of strongly disagree, disagree, neutral, agree, and strongly agree.

A convenience sample of registered nurses was recruited. We included registered nurses with at least one year of work experience. Questionnaires were distributed among 150 nurses in six private, public, and military hospitals to ensure a large and diverse response. The hospitals were: Combined Military Hospital, Lahore; Mayo Hospital, Lahore; Jinnah Hospital, Lahore; Services Hospital, Lahore; Fatima Memorial Hospital, Lahore; and Hearts International Hospital, Rawalpindi. All participants read and signed informed consent forms, which were returned with each completed questionnaire. No monetary or nonmonetary compensation was offered. Forms were personally distributed and collected by two of the authors (Attique R, Asmaa Y). Participants were approached at their workplace during duty hours, and the rationale of the study was explained to them. Those agreeing to participate in the study were given the questionnaire and asked to complete and return it in 10 minutes. Thirty-three nurses declined to complete the questionnaire. Response rate was 81% (122 out of 150). Five forms were discarded because of incomplete or missing data, duplicate entries, or other problems. One hundred and seventeen forms were entered.

## Results

All the nurses in the sample were females. Of the 117 respondents, 34% were working in the military hospital and 66% were from civil settings (private and public hospitals) as shown in Table 1. The respondents had a mean age of 32 (±9.1) years, a mean height of 62 (±2.5) inches (155 cm), and a mean weight of 59.2 (±10.47) kg. Most of the nurses (55.6%) were single. Most respondents (27.8%) had one to five years of work experience. Approximately one-third (31.6%) of the nurses reported experiencing work-related aches/discomfort in the past 12 months.

**Table 1:** Subject demographics and pain experiences

	N (%)
Rank:	
Military	39 (34.5%)
Civil	74 (65.5%)
Work experience:	
<1 year	14 (12.2%)
1-5 years	32 (27.8%)
5-10 years	20 (17.1%)
10-20 years	25 (21.4%)
>20 years	24(20.5%)
Pain experienced in past 12 months:	
Yes	37 (31.6%)
No	80 (68.4%)
Body sites involved:	
Single site	16 (40.0%)
Two sites	9 (22.5%)
>2 sites	15 (37.5%)
Most significant site of pain:	
None	1 (2.5%)
Neck	4 (10.0%)
Shoulder	8 (20.0%)
Upper back	2 (5.0%)
Lower back	13 (32.5%)
Wrist/hand	3 (7.5%)
Elbow	1 (2.5%)
Hips/thighs	2 (5.0%)
Knees	4 (10.0%)
Ankle	2 (5.0%)
First experience of pain:	
Before training	5 (13.2%)
As a student nurse	8 (21.1%)
1-5 years after graduation	4 (10.5%)
5-15 years	14 (36.8%)
>15 years	7 (18.4%)
Management:	
Did nothing	9 (22.5%)
Self-treatment	7 (17.5%)
Professional help	24 (60.6%)

Forty percent complained of pain involving a single site, most commonly the low back (32%). Among the nurses reporting pain, 36.8% had experienced a first episode during the first five to 15 years of their professional service, and 60.6 % had sought medical help.

There was no correlation between number of years of experience (juniors vs. seniors) and work-related ache, pain, discomfort, or injury that lasted for more than three days in the last 12 months (Chi-squared=1.43, p=0.113). There was a significant association between marital status and pain experience in the last 12 months (Chi-squared 3.9, p=0.0001), with a greater proportion of married than single nurses reporting pain. (Table 2)

**Table 2:** Association between marital status and pain experience

Marital status	Pain experienced in last 12 months		Total
	Yes	No	
Married	35	13	48
	72.9%	27.1%	44.4%
Unmarried	38	22	60
	63.3%	36.7%	55.6%

The responses to questions about management and prevention of WRMDs are in Tables 3-4 below.

**Table 3:** Responses of nurses to statements regarding management of WRMDs

For someone with this problem:	Agree (n)	Unsure (n)	Disagree (n)	p-value
Physical activity should be avoided as it might cause harm.	45 (38.5%)	8 (6.8%)	64 (54.7%)	<0.001
These problems usually get better within three months.	44 (37.6%)	20 (17.1%)	53 (45.3%)	0.001
Rest is needed to get better.	110 (94%)	4 (3.4%)	3 (2.6%)	<0.001
Neglecting problems of this kind can cause permanent health problems.	105 (89.7%)	5 (4.3%)	7 (6.0%)	<0.001
These problems are commonly caused by a person’s job.	38 (32.5%)	18 (15.4%)	61 (52.1%)	<0.001

**Table 4:** Responses of nurses to statements regarding perceived causes of WRMDs

Pain is caused by:	Agree (n)	Unsure (n)	Disagree (n)	p-value
Performing the same task again and again (repeatedly)	80 (68.4%)	11 (9.4%)	26 (22.2%)	<0.001
Attending an excessive number of patients in one day	95 (81.2%)	6 (5.1%)	16 (13.7%)	<0.001
Not enough breaks/pauses in one day	78 (66.7%)	14 (12%)	24 (20.5%)	<0.001
Working in awkward and cramped positions	92 (78.6%)	10 (8.5%)	15 (12.8%)	<0.001
Working in the same positions for long periods (standing, bending, sitting)	108 (93.1%)	1 (0.9%)	7 (6.0%)	<0.001
Reaching/working away from your body	75 (64.1%)	17 (14.5%)	25 (21.4%)	<0.001
Prolonged work scheduling (overtime, irregular shifts, length of work day)	86 (73.5%)	4 (3.4%)	27 (23.1%)	<0.001
Inadequate training on injury prevention and posture correction	66 (56.9%)	9 (7.8%)	41 (35.3%)	<0.001

## Discussion

To the best our knowledge and an extensive online literature search, this is the first attempt to document the pattern and perceptions of WRMDs among Pakistani nurses.

We found the overall 12-month-period prevalence rate of WRMDs among our cohort of Pakistani nurses to be 31.6%. This is less than the reported prevalence rates for Australian nursing students (80.0%) [12] and nurses in Japan (85.5%) [14], Iran (95%) [17], mainland China (90%) [18], India (41%) [19], Nigeria (88.4%) [10], and Estonia (84%) [13]. The low prevalence rate among Pakistani nurses can be attributed to the following factors. The shifts of nurses in our sample ranged from six to eight hours per day. The workload of patient care is less, as Pakistani nurses usually do not lift or transfer patients, particularly male patients. This aspect of direct patient care is often handed over to male nursing assistants. The increased lifting work of nursing aides might be a reason for the reported high prevalence of  WRMDs among nursing aides as compared to registered nurses [11,20-21]. Cultural factors can also play a role [22]. In Pakistan, women—including nurses—tend not to report problems like pain and discomfort, and tend to suffer in silence. They also tend to downplay their suffering. Lack of definition/subjectivity of terms is another factor in low prevalence. Most nurses in our study, when questioned, did not have any idea whether their pain was significant (saying, for example, that it was just a little backache and it happened all the time).

In our study, the most commonly affected site was the lower back (32%), followed by the shoulders (20%), knees (10%) and neck (10%). This is consistent with the pain patterns reported in Turkish nurses (69% back, 54% shoulders) [11], Australian nurses (59.2% lower back, 34.6% neck) [12], Nigerian nurses (50% lower back, 27.5% shoulders) [10,23], and Indian nurses (48.2% lower back, 34.6% shoulders) [19]. However, in Japanese nurses, incidence of shoulder pain and lower back pain was almost the same (71.3%) and more common than neck pain (54.7%) [14]. Similarly, in Brazilian nurses, pain in the upper back, neck, or shoulders (57.1%) was more prevalent than pain in the lower back (53.9%) [20].

Among people in the healthcare industry, nurses have a relatively high prevalence of WRMDs [23]. The reasons are thought to be high workload, long work hours, and lack of awareness among nurses regarding prevention of WRMDs [10]. WRMDs are thought to be more common among married nurses, perhaps because they have an additional role of home and child care [20]. Duration of work experience and age have been associated with WRMDs in some studies [12,15,21]; however, we did not observe any relationships between these factors and prevalence of WRMDs.

In a study of rural Australian nursing students, male nursing students had a higher prevalence of WRMDs than female nursing students [12]. In that population, males were more actively involved in manual handling patients, as is the case in Pakistani culture. However, as our study was limited to female nursing staff only, this factor could not be explored. The male lifting role might be a reason for low prevalence of WRMDs among Pakistani nurses.

Of the nurses in our study who reported symptoms, 60.6% sought help from health professionals. This is higher than Greek and Dutch nurses (50%) [23]. This is probably because nurses in Pakistan can often consult a physician directly without a referral.

In our study, 81.2% of the nurses expressed agreement with the statement that attending an excessive number of patients per day will lead to worsening of musculoskeletal pain. Manual handling of patients has been shown to be a risk factor for low back pain. In Japan, nurses who performed manual handling of patients were 2.07, 2.59, and 11.97 times more at risk, respectively, for shoulder, low back, and other WRMDs [14]. The comparison among Greek and Dutch personnel done by Alexopoulos et al. also revealed increased prevalence of lower back pain among Greek personnel due to increased physical load [23]. June et al. reported 90.3% of intensive care unit (ICU) nurses having low back pain at least once a month, which indicates high workload in ICUs [24]. Korean nurses working in ICUs and surgical wards also reported an increased incidence of WRMDs possibly due to heavy manual handling of patients [25]. Lifting and otherwise handling patients, as nurses often must do, generate biomechanical stress, which is an important factor in shoulder and back pain [12].

The most frequently identified factors contributing to the development of WRMDs were working in the same position for prolonged periods (93.1%), working in an awkward or cramped position (78.6%), and reaching or working away from one’s body (64.1%) as shown in Table 4. A study done on Latin American nurses identified 1.37 times more risk of neck, shoulder, upper back, and lower back pain in personnel exposed to physical demands (heavy lifting, poor posture of back, and repetitive gestures) [20]. Workshops on injury prevention and posture correction can help address such problems, but they are undervalued. Long sitting hours, prolonged standing, repetitive movements, continuous bending, lifting/transferring dependent patients, cramped and cluttered workplace, and understaffing are the suggested risk factors for WRMDs; however, the mechanism is yet to be established [23].

Discussion with nursing students and nursing staff at hospitals where we conducted our study indicated that there was no formal training in ergonomics, maintenance of adequate posture, and the value of micro-breaks in reducing WRMDs either at the under- or postgraduate level. To improve nurses' understanding regarding WRMDs and to reduce the incidence of WRMDs, workshops and seminars should be conducted regularly. Training sessions should be held to familiarize nursing students with the concept of ergonomics [23].

### Limitations

The cross-sectional design of this study limits inferences about causality and temporality. The use of a self-administered questionnaire can lead to information bias and recall bias. We had a relatively small sample obtained using a convenience sampling approach. Therefore, the results might not be generalizable to nurses working in different work environments and different parts of the country. Nurses working in different departments have different workloads and duty hours. Some departments, such as ICUs, are more demanding than others. We did not stratify nurses by department. While English is the language of instruction in Pakistan, it is not the language of daily and general communication for the majority of people, including nurses. A future study with a validated Urdu translation may add value to this work.

## Conclusions

About one-third of Pakistani nurses in this cohort reported work-related musculoskeletal disorders with the low back being most commonly affected. The most common contributing factors identified were working in the same position for prolonged periods, working in an awkward or cramped position, and reaching or working away from one’s body. This suggests that WRMDs are preventable disorders. There is a need to increase awareness regarding ergonomics and posture maintenance to reduce WRMDs and improve patient care. This can be achieved by workshops and seminars on ergonomics and WRMDS.
